# Successful Emergency Stenting of a Visceral Branch Prior to Central Aortic Repair in Type A Aortic Dissection with Mesenteric Malperfusion: A Case Report

**DOI:** 10.70352/scrj.cr.25-0136

**Published:** 2025-06-18

**Authors:** Sho Akita, Akinori Tamenishi, Yasumoto Matsumura, Kunihiro Maruyama, Jun Ito

**Affiliations:** 1 Department of Cardiac Surgery, Yokkaichi Municipal Hospital, Yokkaichi, Mie, Japan; 2 Department of Radiology, Yokkaichi Municipal Hospital, Yokkaichi, Mie, Japan

**Keywords:** type A aortic dissection, superior mesenteric artery malperfusion, endovascular stenting, central aortic repair, mesenteric ischemia, staged approach

## Abstract

**INTRODUCTION:**

Stanford Type A acute aortic dissection (AAD) complicated by mesenteric malperfusion has a mortality rate exceeding 60%. Conventional immediate central aortic repair may be inadequate in such complex cases. Emerging evidence suggests that a staged approach may improve outcomes.

**CASE PRESENTATION:**

A 71-year-old male presented with acute chest pain and was diagnosed with Stanford Type A AAD extending to the abdominal aorta, with superior mesenteric artery (SMA) dissection leading to intestinal ischemia. To restore intestinal perfusion, emergency endovascular SMA stenting was performed as the initial intervention, followed by ascending aorta and total arch replacement using the frozen elephant trunk technique 12 hours later. The patient recovered without complications and was discharged ambulatory on postoperative day 20.

**CONCLUSIONS:**

This case highlights the efficacy of a staged approach prioritizing mesenteric revascularization before central aortic repair in AAD complicated by visceral malperfusion. By first addressing end-organ ischemia, we potentially mitigated the risk of irreversible bowel necrosis while enabling subsequent central aortic repair. Our experience adds to the growing body of evidence supporting individualized, pathophysiology-guided treatment strategies for this challenging clinical scenario.

## INTRODUCTION

Mesenteric ischemia complicates 3%–5% of Stanford Type A aortic dissection (AAD) cases and carries an in-hospital mortality rate exceeding 60%.^1,2)^ Conventional management through immediate central aortic repair may be insufficient when complicated by visceral malperfusion, with mortality rates significantly elevated in such clinical scenarios.^3)^

The pathophysiology of malperfusion typically involves either dynamic obstruction from mobile intimal flaps or static obstruction from true lumen compression.^4)^ Understanding these mechanisms is crucial for determining optimal intervention strategies. Recent evidence suggests that a staged approach—with endovascular intervention preceding central repair—may improve outcomes, particularly in cases of static obstruction.^5)^

Early reperfusion of affected visceral arteries, especially the superior mesenteric artery (SMA), is essential for preventing irreversible bowel necrosis. However, the optimal sequence and timing of interventions remain debated, particularly regarding whether to prioritize central repair or address end-organ malperfusion first.^6)^

We present a case of successful management of AAD with concurrent SMA malperfusion through a staged approach, highlighting the rationale for our treatment decisions and the favorable outcomes achieved.

## CASE PRESENTATION

A 71-year-old male with a history of hypertension and active tobacco use (20 pack-years) presented to our emergency department with acute-onset chest tightness radiating to severe interscapular pain. On examination, he was hemodynamically stable with a blood pressure of 142/85 mmHg, heart rate of 92 bpm, respiratory rate of 18 breaths per minute, and oxygen saturation of 96% on room air. The patient reported mild nausea and vomiting but denied abdominal pain. Laboratory testing revealed mild leukocytosis (11520/μL), normal troponin levels, and a lactate concentration of 11 mg/dL. Aspartate transaminase was 19 IU/L, alanine transaminase 14 IU/L, and creatine kinase within normal range, with no findings suggestive of intestinal ischemia.

An initial electrocardiogram showed precordial ST-segment depression in leads V2–V4. Transthoracic echocardiography revealed a left ventricular ejection fraction of approximately 60%, with no regional wall motion abnormalities and no significant valvular disease.

**Fig. 1:**
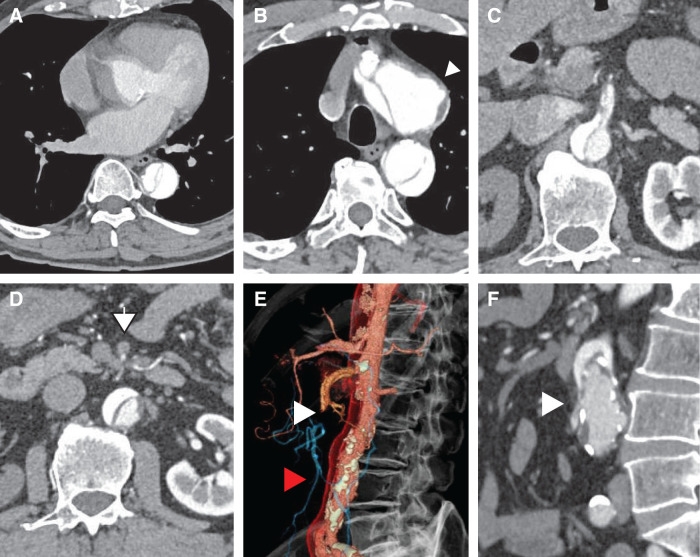
CT findings in preoperative aortic dissection. (**A**) Thrombosis is present in the false lumen of the ascending aorta. (**B**) A saccular aneurysm is visible in the distal aortic arch. (**C**) Dissection involves the SMA origin, demonstrating a characteristic double-barrel appearance with blood flow present in both true and false lumens. (**D**, **E**) Complete occlusion of the main SMA trunk (white arrowhead); distal SMA is opacified via collaterals from the IMA (red arrowhead). (**F**) Calcified stenosis at the IMA ostium with reduced contrast enhancement. IMA, inferior mesenteric artery; SMA, superior mesenteric artery

Urgent computed tomography angiography (CTA) confirmed Stanford type A aortic dissection with retrograde extension from a primary intimal tear in the proximal descending aorta (DeBakey type IIIb), extending into the abdominal aorta (**Fig. 1**). The ascending aortic false lumen was completely thrombosed, with no pericardial effusion or signs of rupture. A 48-mm saccular aneurysm was identified at the distal aortic arch near the ligamentum arteriosum. The false lumen was thrombosed proximally but remained patent from the descending aorta distally.

Dissection involved the origin of the SMA, with complete collapse of the true lumen (**Fig. 1**). Although the celiac trunk origin showed some degree of stenosis, antegrade flow was preserved. The inferior mesenteric artery (IMA) remained patent, although its ostium was calcified and showed poor contrast enhancement. The distal SMA was opacified, and radiological assessment suggested that mesenteric perfusion was primarily maintained via collateral flow from the IMA. No signs of bowel wall hypo-enhancement or pneumatosis were observed. The superior mesenteric vein (SMV) measured 13 mm and the SMA 10 mm, yielding an SMV/SMA ratio of 1.3. Right renal artery hypoperfusion was noted, though no evidence of bowel wall edema was seen on imaging.

### Surgical management

#### Initial management

Despite preserved hemodynamic stability and normal serum lactate levels, early imaging confirmed SMA obstruction. A multidisciplinary consensus was reached to prioritize urgent endovascular therapy (EVT) to restore mesenteric perfusion.

**Fig. 2:**
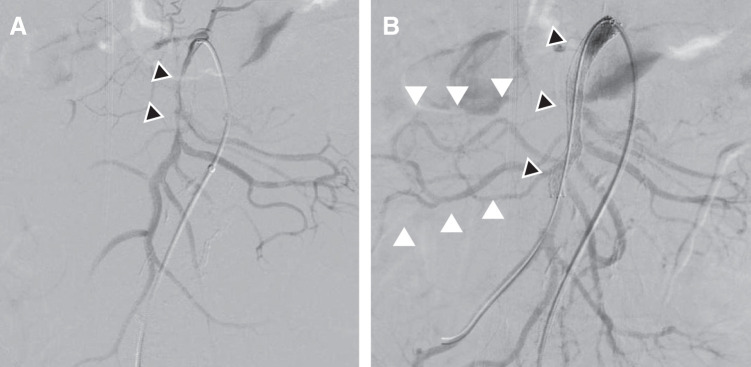
(**A**) Angiography showing critical stenosis of the SMA (black arrowheads), with near-total occlusion of the true lumen. (**B**) Post-intervention angiography after self-expandable stent (7 mm × 8 cm) deployment (black arrowheads), showing significant improvement in true lumen diameter and antegrade blood flow (white arrowheads). SMA, superior mesenteric artery

Via percutaneous left femoral artery access, selective SMA angiography confirmed flow-limiting dissection at the SMA origin (**Fig. 2A**). A 7 mm × 8 cm self-expandable stent (EverFlex, Medtronic, Dublin, Ireland) was deployed, resulting in immediate restoration of antegrade flow with excellent distal perfusion (**Fig. 2B**).

Post-procedural monitoring in the intensive care unit revealed no elevation in serum lactate and resolution of gastrointestinal symptoms. The patient remained stable without signs of progressing organ dysfunction.

#### Staged central repair

SMA stenting was completed at night, and the patient remained hemodynamically stable without signs of rupture or tamponade. After multidisciplinary discussion, we decided to perform central repair the following morning, approximately 12 hours later.

This short delay allowed us to monitor for improvement in mesenteric perfusion and to ensure there were no signs of reperfusion injury. We believed that confirming the patient's metabolic stability before major surgery was important for improving surgical safety and outcomes.

Twelve hours after endovascular treatment, with stable vital signs and improved laboratory data, the patient underwent central aortic repair via median sternotomy under cardiopulmonary bypass. Dual arterial cannulation (left axillary and femoral arteries) and systemic cooling to 25°C were used. Intraoperative findings confirmed a proximal descending intimal tear and a saccular aneurysm in the distal arch, consistent with preoperative imaging.

Total arch replacement was performed using a quadrifurcated collagen-coated woven polyester graft (J graft, Japan Lifeline, Tokyo, Japan; 26-mm trunk with 11-mm, 9-mm, and 9-mm branches), with a Frozen Elephant Trunk (Frozenix, 29 mm × 12 cm, Japan Lifeline) deployed into the descending thoracic aorta.

Intraoperative transesophageal echocardiography following weaning from cardiopulmonary bypass revealed anterior wall hypokinesis. Concern for left main trunk (LMT) stenosis due to hematoma compression prompted concomitant coronary artery bypass grafting (CABG) with a saphenous vein graft to the left anterior descending artery (SVG-LAD).

Operative parameters included total operative time of 6 hours 13 minutes, cardiopulmonary bypass time of 2 hours 47 minutes, aortic cross-clamp time of 77 minutes, and lower body circulatory arrest duration of 41 minutes. Hemostasis required transfusion of 12 units each of leukocyte-depleted packed red blood cells and fresh frozen plasma, as well as 20 units of platelet concentrate.

#### Postoperative course

**Fig. 3:**
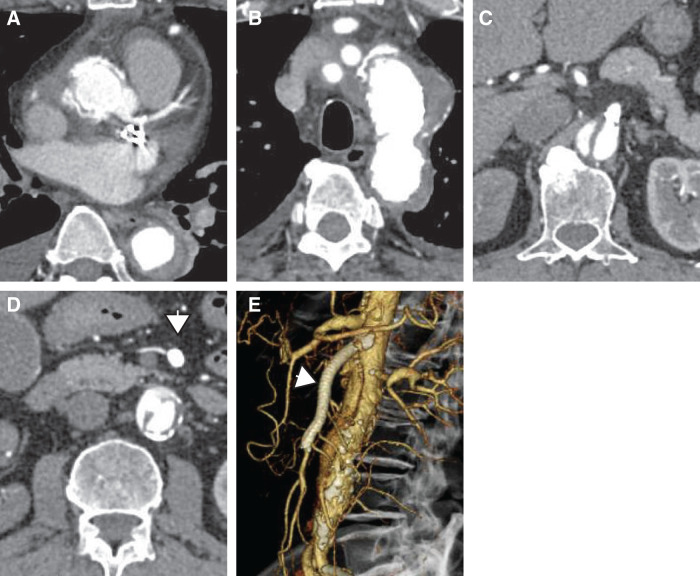
CT findings following TAR with FET. (**A**) The ascending aorta has been replaced with a prosthetic graft. (**B**) The saccular aneurysm in the distal arch shows complete thrombosis. (**C**) Blood flow to the SMA origin is restored through the true lumen. (**D**, **E**) The main trunk of the SMA demonstrates patent flow (white arrowhead). FET, frozen elephant trunk; SMA, superior mesenteric artery; TAR, total arch replacement

The patient was extubated on postoperative day (POD) 2 and transferred out of intensive care on POD 4. Postoperative contrast-enhanced CT demonstrated proper graft positioning and sustained SMA patency (**Fig. 3**). Both antegrade flow in the LMT and graft patency to the LAD were confirmed. The patient resumed oral intake on POD 5 without abdominal complaints, and bowel function normalized by POD 7. He was discharged ambulatory on POD 20 and remained well at 1-month follow-up, with persistent SMA stent patency and no signs of dissection progression.

## DISCUSSION

Our therapeutic strategy was informed by three key considerations:

First, the static nature of the SMA obstruction necessitated immediate anatomic correction. In such cases, central aortic repair alone is insufficient, as the compressed true lumen at the branch origin may persist despite proximal intervention.^5,6)^ This contrasts with dynamic obstruction, where central repair may relieve malperfusion by decompressing the false lumen.

Second, staging allowed assessment of visceral viability after revascularization while minimizing the risk of ischemia-reperfusion injury during cardiopulmonary bypass. According to the International Registry of Acute Aortic Dissection (IRAD), patients with malperfusion have higher mortality when undergoing immediate central repair compared with those treated with initial EVT followed by delayed surgery.^1)^ In our case, the SMA was completely occluded and visualized only via retrograde flow through the IMA, whose origin was severely stenotic due to calcification. Although bowel ischemia was not evident on imaging, proceeding with total arch replacement without prior SMA revascularization could have led to ischemic injury during hypothermic circulatory arrest. These factors supported our decision to prioritize revascularization.

Third, endovascular SMA stenting allowed rapid perfusion restoration with minimal physiological disruption. Compared with open procedures, percutaneous intervention offers reduced blood loss, shorter procedure time, and decreased systemic inflammatory response—critical benefits in acutely ill patients.^7)^

Recent reports advocate staged management for type A aortic dissection with malperfusion. Yang et al. reported improved outcomes and a decrease in operative mortality from 21% to 11% with this approach.^5)^ Similarly, Huang et al. demonstrated reduced mortality (54.55% vs. 18.52%, p = 0.047) with a staged strategy of initial endovascular revascularization followed by delayed central repair.^8)^

However, in cases of visceral ischemia with hemodynamic instability due to cardiac tamponade or high rupture risk from a patent false lumen, the optimal sequencing of central aortic repair versus visceral branch intervention remains controversial. Our patient presented with a DeBakey type IIIb dissection with thrombosed proximal false lumen, indicating low rupture risk. Conversely, DeBakey type I dissections originating in the ascending aorta carry higher rupture risk and worsening malperfusion, typically requiring immediate central repair. In cases of severe intestinal ischemia, concurrent visceral branch intervention may be necessary.

When hybrid operating rooms or endovascular support are unavailable during emergencies, open surgical exposure of the SMA becomes essential. Direct surgical revascularization of the SMA trunk has shown favorable outcomes. Cardiac surgeons should therefore be familiar with abdominal exposure and vascular control techniques for the SMA to perform these combined procedures effectively.^9)^

Treatment strategies should be individualized based on malperfusion severity across multiple organs beyond rupture risk. For example, coronary malperfusion may necessitate adjunctive percutaneoous coronary intervention (PCI) or CABG.^10,11)^ In this case, dissection extended to the aortic root, with the thrombosed false lumen reaching the proximal LMT. Despite this, echocardiography demonstrated preserved wall motion, allowing for elective central repair. Postoperatively, the patient developed anterior wall hypokinesis requiring saphenous vein grafting to the LAD. This complication likely resulted from LMT compression caused by either surgical glue entering the false lumen after thrombus removal or hematoma formation near the proximal anastomosis. When the aortic root is involved, meticulous central repair is crucial to prevent such complications.

## CONCLUSIONS

This case demonstrates that staged management involving emergency SMA stenting followed by delayed central aortic repair offers a viable strategy for acute type A aortic dissection complicated by mesenteric malperfusion. This approach addresses both the urgency of restoring visceral perfusion and the complexity of definitive aortic repair. Further multicenter studies are warranted to validate this strategy and refine patient selection criteria.

## DECLARATIONS

### Funding

Not applicable.

### Authors’ contributions

All authors contributed to the treatment plan, manuscript drafting, and critical revision. All authors read and approved the final manuscript.

### Ethics approval and consent to participate

Informed consent to participate in this study was obtained from the patient.

This work does not require ethical considerations or approval.

### Consent for publication

Informed consent for publication of this case report was obtained from the patient.

### Competing interests

The authors declare that they have no competing interests.
